# Methods of Rescue Work

**Published:** 1898-07-16

**Authors:** 


### Modern Sociology.

### METHODS OF RESCUE WORK.

One of the most significant notes of the great progress in humanity which has characterised the last half century, has undoubtedly been in the impulse given to systematic efforts for the rescue of fallen women. The principle which has brought life to the movement is admirable, maintaining, as it does, that no higher mission could be found for pure-minded women than the devotion of themselves to the work of raising their unhappy sisters from the depths of infamy to blameless lives of true reform. And much has been accomplished, although the teeming midnight streets of London and all large cities might indicate that the measure of success obtained has been but as a drop in the ocean of vice. Still, though the record of the last fifty years can show, perhaps, only some hundreds of hopeful cases, where thousands should have been numbered if any perceptible impression had been made on the gigantic evil, there is now a very widespread organisation in this country for the attainment of that desirable object. It is because we believe that the work might be infinitely more efficacious if the methods employed were in some respects different, that we touch on this unattractive subject. A certain amount of effort in this direction has always existed in religious centres of varying denominations, but the new impulse which galvanised it into more energetic life in the earlier part of this century emanated almost exclusively from the High Church party. As a result, the great majority of the Homes which have been established for the reception of so-called penitents are conducted by the various Sisterhoods now existing in connection with it. The system, therefore, on which existence is moulded in these high-class penitentiaries is in strict accordance with the principles of what is technically termed the "religious life"—that is, the rules and arrangements are practically such as would obtain in a nunnery. If the young women who are invited to enter there were really penitents, in the true sense of the word, the spiritual education they receive in these Homes might tend to give consistency and value to a regret for their evil lives, founded on a true recognition of its moral criminality. But penitence of this nature, though it may be engendered ultimately by good influences brought to bear on them, is scarcely ever the motive which induces them to seek a shelter from life on the streets. It is, in fact, almost impossible that it should exist in the case of those who have adopted this shameful trade, for all who have had an opportunity of judging, can testify that the habitual practice of immorality, more than any other species of wrong-doing, has power to deaden the conscience and destroy even the faculty of distinguishing between right and wrong. Many reasons may induce women of this description to enter a Home—perhaps ill-treatment from the men with whom they associate, weak health, or only a sickening weariness of the uncertainties of their calling, in which, with much meretricious glitter and pleasure, there is also often great hardship and suffering. Sometimes they are persuaded against their will by those who wish them well; but, whatever may be the cause of their entrance, it surely stands to reason that the change from a life of the most utter lawlessness and independence, to which they themselves give the name of a "gay life," into the solemn observances and rigid discipline of a convent is too violent a contrast to be endurable to them. There they find themselves subjected to periods of enforced silence, to constant religious services, and complete seclusion—even sometimes to what are called "retreats," which mean two or three days spent wholly in the chapel listening to sermons and singing hymns; and while all this might be salutary enough if they were really repentant sinners, it is enforced on those who are for the most part but the willing victims of self-chosen licentiousness and pleasure seeking. These penitentiaries are known among the women of the class they desire to benefit as "two years' Homes," because their conductors hold, rightly enough, that at least two years' training is absolutely required to give any hope of a real reform, and therefore every girl who is brought for admission there, is made to understand that she must be prepared to remain within the walls for that length of time. As a consequence it will be within the experience of all who have attempted the exceptionally difficult work of rescue, that the idea of a two-years' detention is strongly resisted by these unhappy women, and they turn from the suggestion with invincible repugnance. We must beg our readers to understand that we do not for a moment wish to depreciate the good work done by the admirable women who conduct these institutions in all cases where there is a genuine desire of reform, or the kind treatment and devoted service they give to their unfortunate charges; our object is simply to propose a remedy for some of what we consider to be the mistakes of the system, with a view to their greater efficiency. We hold strongly that every one of these Homes ought to have a preliminary Refuge affiliated with it, which the poor girls should be invited first to enter—nothing at all being said of a "two years' Home"—while they should be emphatically made to understand that there is no obligation on them whatever to remain for any stated period, and that they will be perfectly free to leave it whenever they please, be it in a month, or a week, or even a day, just as they may themselves decide. During the time that they inhabit this temporary Home they ought to be subjected only to the very simplest rules consistent with their safety, and should be be made to feel that it is merely a shelter for them—from vice, from temptation, and danger—where every kindness will be shown them, every reasonable comfort supplied, and where they will be received at any moment, night or day, with no promises asked and nothing required of them beyond decorous and quiet conduct while they choose to remain. Every effort should be made to induce them to enter on a life of real reform, the ultimate object held in view by the managers of the Refuge being simply the final disposal of their unhappy charges in such a manner as shall be most likely to procure this result. Often, indeed, these poor women may be found to be in a state of health which renders it impossible for them to be retained even in the Refuge, and these must be removed to hospitals with the understanding that they will be again received when cured; in accordance with the primary rule of the shelter—that every woman who applies for admission shall be welcomed at once under its roof without question or hesitation.

